# Submersion, accidental hypothermia and cardiac arrest, mechanical chest compressions as a bridge to final treatment: a case report

**DOI:** 10.1186/1757-7241-17-7

**Published:** 2009-02-20

**Authors:** Hans Friberg, Malin Rundgren

**Affiliations:** 1 Department of Anesthesia and Intensive Care, Lund University Hospital, Lund, Scania, Sweden

## Abstract

Three young men were trapped in a car at the bottom of a canal at two meters depth, after losing control of their vehicle. They were brought up by rescue divers and found in cardiac arrest. One of three patients had return of spontaneous circulation (ROSC), at 47 min after the accident. This sole survivor had the longest submersion time of the three and he received continued mechanical chest compressions during transportation to the hospital. His temperature at admission was 26.9°C, he was rewarmed to 33°C and kept there for 24 h, followed by continued rewarming to normothermia. On day three, he woke up from coma and was discharged from the intensive care unit after one week. At follow-up six months later, he had a complete cerebral recovery but still had myoclonic twitches in the lower extremities. A mechanical device facilitates chest compressions during transportation and may be beneficial as a bridge to final treatment in the hospital. We recommend that comatose patients after submersion, accidental hypothermia and cardiac arrest are treated with mild hypothermia for 12–24 h.

## Background

Submersion with cardiac arrest is a great challenge to our prehospital rescue teams. First, rescue divers must bring the victims to the surface, followed by cardiopulmonary resuscitation (CPR) and transportation to a hospital. Submersion time, water temperature and prompt resuscitation seem to be crucial factors for outcome, and so do age and time for the rescue team to arrive on scene [1,2]. Submersion in cold water and subsequent accidental hypothermia may be beneficial [3,4], if circulation can be restored. There are no randomized, controlled trials (RCT) evaluating care of submersion patients since, luckily, the victims are few. We report a case of successful resuscitation after using mechanical chest compressions in a patient with cardiac arrest due to hypothermia caused by submersion.

## Case presentation

A cold Saturday night in mid March, the driver of a car lost control and the car went over the barrier and through the ice into a canal. The accident occurred in a densely populated area in southern Sweden and was observed by several people. Rescue divers and ambulance staff were immediately notified and were on the scene 11 min later. Within another 10 min, three young men, trapped in the backseat of the car at a depth of two meters, had been rescued; all three were pulseless with asystolic cardiac arrest. CPR was immediately initiated in all three, one was transported to the local hospital with ongoing manual chest compressions but never had return of spontaneous circulation (ROSC), and was eventually declared dead. Two patients were transported to Lund University Hospital with ongoing CPR (patient 1 and 2), a 15 min drive away.

### Patient 1

A 27-year old male was the second one to be brought up by the divers. He was transported to hospital with ongoing manual chest compressions and mask ventilation. Out-of-hospital intubation failed and he was intubated on arrival in the emergency room (ER), approximately 40 min after the accident. At this time, the patient still had asystole and mechanical chest-compressions were started (LUCAS^®^, Jolife AB, Lund, Sweden). The patient presented with an initial tympanic temperature of 29.0°C and a profound combined metabolic and respiratory acidosis with a pH of 6.7 (Table 1). Initial treatment included multiple doses of atropine and epinephrine, buffer, warm fluids and controlled ventilation. Cardiopulmonary by-pass assistance (CPB) was considered but both on call teams were occupied. CPR with LUCAS^® ^and warm fluids continued for another 45 minutes without ROSC, why resuscitation attempts stopped 90 minutes after the accident. Central temperature reached 33°C and the patient was declared dead. An autopsy in the Department of Forensic Medicine revealed no major injuries.

**Table 1:** Patient characteristics (all time measures in min).

	**Patient 1**	**Patient 2**
Sex	male	male

Age (years)	27	34

Rescue team on scene	11	11

Submersion time	20	21

Time to CPR	21	22

Initial rhythm	asystole	asystole

Chest compressions	manual	mechanical

Secured airway	in hospital	in ambulance

Time to ROSC	N/A	47

Outcome 6 months	dead	alive

Initial temperature	29.0°C	27.9°C

Initial pH (α-stat)	6.7	6.8

### Patient 2

**Figure 1:**
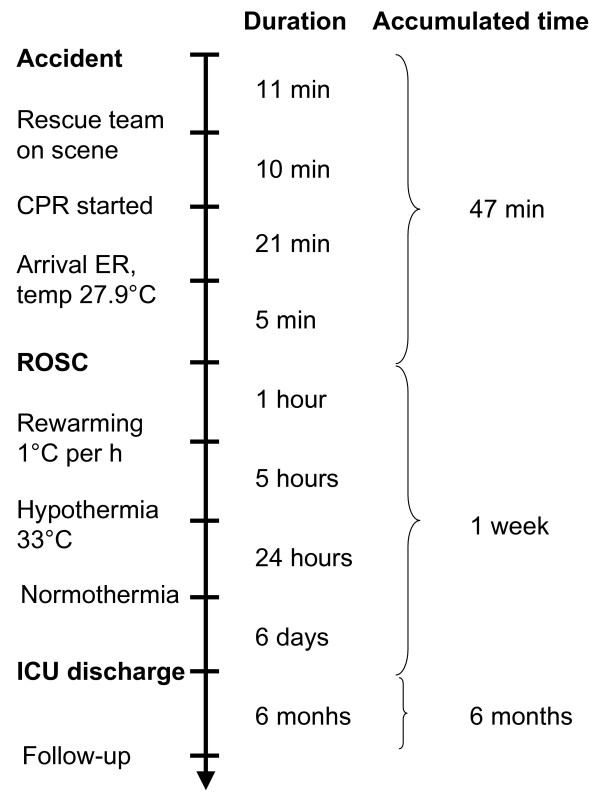
**Duration of interventions and accumulated time after submersion, accidental hypothermia and cardiac arrest in one surviving patient (patient 2)**.

A 34-year-old male was the last person to be brought up by the rescue-divers, approximately 21 min after submersion. The initial rhythm was asystole and mechanical chest compressions, using the LUCAS^® ^device, were started on scene and continued without interruption en route to the hospital. The patient was initially mask ventilated but was intubated in the ambulance during ongoing mechanical chest compressions, approximately 30 min after the accident. On arrival in the ER, 42 min after the accident, he still had asystole and the tympanic temperature was 27.9°C. He had a severe combined metabolic and respiratory acidosis with a pH of 6.8 (Table 1). Following continued CPR and administration of atropine, adrenaline, buffer and warm fluids in the ER, he eventually had ROSC at approximately 47 min after the accident. A computer tomography (CT) of the head, neck, thorax and abdomen revealed no major injuries and the patient was brought to the intensive care unit (ICU) with stable circulation. Cardiopulmonary by-pass assistance was again considered, but still unavailable, why an IcyCath^® ^catheter (Alsius Corp., CA, USA) was placed in the femoral vein for rewarming and temperature control. Temperature was increased 1.0°C per hour to 33°C, and maintained for 24 h, followed by controlled rewarming to normothermia (0.5°C per hour) [5]. An acute respiratory distress syndrome (ARDS) developed and repeated bronchoscopies revealed a general glassy oedema. Still, the patient improved and at normothermia, sedation was reduced. Two and a half days after the accident he regained consciousness and could respond adequately, and was extubated on the seventh day. The brain damage markers S-100B and neuron specific enolase peaked at 12 h with values of 0.31 and 21.3 ug/L respectively (reference intervals < 0.04 ug/L and < 12.5 ug/L). Routine amplitude integrated EEG-monitoring (aEEG) showed a continuous pattern from the start and onwards, which is a good prognostic sign for cardiac arrest survivors [6]. Severe myoclonic seizures developed on day three that only partly responded to treatment with bensodiazepines. After eight days in the ICU, he was transferred to an ordinary ward and eventually to a rehabilitation facility. He was discharged after two months and at follow up, 6 months after the accident (Figure 1), he had recovered fully except for sporadic myoclonic twitches in the lower extremities. He had no memory for the time surrounding the accident and was in cerebral performance category (CPC) 1 [7].

In this report, three formerly healthy young men were rescued with pulseless asystole and severe accidental hypothermia after submersion in cold water; one regained spontaneous circulation and eventually recovered fully. All three were treated by the same prehospital team and the only survivor was the last one to be brought up by the rescue divers. The two patients who were taken to our hospital both had initial mask ventilation, both were intubated with approximately 10 min interval, followed by controlled ventilation. One had initially manual (patient 1) and the other continued mechanical (patient 2) chest compressions.

Why only patient 2 regained circulation can only be speculated on; one reason may be that his airway was secured at an earlier time than patient 1. The potential benefit of younger age in cases of accidental hypothermia and submersion has been addressed [8], but age did not differ between the survivor and the non-survivors in this report. Another reason may be that early and uninterrupted mechanical chest compressions in our survivor made a difference. There are experimental studies and case reports supporting a beneficial effect of mechanical chest compressions [9,10], but there are no RCTs supporting its use [11-13]. However, it has been shown that "hands-off time" is shorter and compression quality is improved when a mechanical device is used during transportation [14,15]. On arrival in the ER, both patients had a severe combined acidosis, a marker of a bad outcome [16]. Once ROSC was established in our survivor and a CT-scan had excluded major trauma, controlled rewarming to 33°C and therapeutic hypothermia for 24 h was performed, using a femoral catheter and an external temperature control device. The use of CPB in assisting circulation and for controlled rewarming has been recognized as the method of choice in this situation [17,18], and was also considered in our patient(s). Due to a limited 24 h access to CPB capacity, even in a university hospital, an intravenous catheter and an external temperature control device may be used as an alternative method for controlled rewarming in patients with ROSC. In our patient, rewarming was stopped at 33°C and the temperature kept stable for 24 h, which is in compliance with existing guidelines, stating that therapeutic hypothermia may be considered for patients with initial non-shockable rhythms [19]. A similar case with accidental hypothermia (without submersion), cardiac arrest and prolonged resuscitation including mechanical chest compressions during transportation, was recently highlighted [20].

## Conclusion

Submersion victims with accidental hypothermia and cardiac arrest should be treated according to existing CPR guidelines. A mechanical chest compression device facilitates chest compressions during transportation and may be beneficial as a bridge to final treatment in the hospital. Accidental hypothermia must be corrected, if possible in a hospital with CPB capacity. We recommend that rewarming should be stopped at 33°C in comatose patients, followed by 12–24 h treatment before continued rewarming to normothermia.

## Consent

Written informed consent was obtained from the surviving patient for publication of this case report, and from next of kin of the two casualties. A copy of the written consent is available for review by the Editor-in-Chief of this journal.

## Competing interests

The authors declare that they have no competing interests.

## Authors' contributions

Both authors contributed equally to data retrieval and writing of this manuscript
